# Risk factors associated with failure of total ankle arthroplasty: a nationwide cohort study

**DOI:** 10.1038/s41598-021-82674-7

**Published:** 2021-02-03

**Authors:** Dong Hun Suh, Kyungdo Han, Jin Woo Lee, Hak Jun Kim, Bongsung Kim, Bong Mo Koo, Hak Kyu Kim, Gi Won Choi

**Affiliations:** 1 Department of Orthopaedic Surgery, Korea University Ansan Hospital, 123, Jeokgeum-ro, Danwon-Gu, Ansan-si, Gyeonggi-do 15355 Republic of Korea; 2 Department of Statistics and Actuarial Science, Soongsil University, Seoul, Republic of Korea; 3 Department of Orthopaedic Surgery, College of Medicine, Yonsei University, Seoul, Republic of Korea; 4 Department of Orthopaedic Surgery, Korea University Guro Hospital, Seoul, Republic of Korea

**Keywords:** Diseases, Risk factors

## Abstract

We conducted a nationwide population-based cohort study to identify the risk factors associated with failure of total ankle arthroplasty (TAA). We included 2,914 subjects who underwent primary TAA between January 1, 2010, and December 31, 2016, utilizing the database of the Korean National Health Insurance Service. Failure of TAA was defined as revision TAA or arthrodesis procedures. An increased risk of TAA failure was observed in the < 65 age group versus the ≥ 75 age group [adjusted hazard ratios (aHR) 2.273, 95% confidence interval (CI) 1.223–4.226 in the 60–64 age group; aHR 2.697, 95% CI 1.405–5.178 in the 55–59 age group; aHR 2.281, 95% CI 1.145–4.543 in the 50–54 age group; aHR 2.851, 95% CI 1.311–6.203 in the < 50 age group]. Conversely, the ≥ 65 age group displayed no increase in the risk of TAA failure. The risk of TAA failure was increased in the severely obese group with body mass index (BMI) of ≥ 30 kg/m^2^ versus the normal BMI group (aHR 1.632; 95% CI 1.036–2.570). This population-based longitudinal study demonstrated that age < 65 years and BMI of ≥ 30 kg/m^2^ were associated with increased risk of TAA failure.

## Introduction

Currently, The surgical treatment options for end-stage ankle arthritis are ankle arthrodesis (AA) or total ankle arthroplasty (TAA)^1,2^. Although, AA has traditionally been the gold standard for end-stage ankle arthritis, TAA has recently shown promising results becoming an accepted treatment option^3–6^. Therefore, the popularity of TAA has rapidly increased in recent years^7–9^. TAA provides comparable clinical outcomes and improved function compared with AA but reoperation rates are higher in TAA^3,10^. reoperation rate of TAA was 17% in a study with a mean follow-up of 5.5 years and 20.5% in a systematic review^11^. Registries from Europe, New Zealand, and Australia report failure rates varying from 7.1 to 19% at 5 years and 24% to 31% at 10 years^12–17^. High reoperation rate is the predominant disadvantage of TAA compared with AA.

Numerous studies have investigated the risk factors associated with failure of TAA, and the identified risk factors include age, body mass index (BMI), diabetes, rheumatoid arthritis, smoking, and preoperative deformity^5,18–24^. However, majority of these studies have been limited by a small sample size, and there have been mixed results concerning the effect of age, BMI, diabetes, and rheumatoid arthritis on failure of TAA, with some studies reporting an increased risk of failure while other report no effects.

Consequently, this study aimed to identify the risk factors associated with failure of TAA by utilizing the data derived from the entire South Korean population.

## Methods

### Data sources

We used a database provided by the South Korean National Health Insurance Service (NHIS), which is a single mandatory health insurance system covering 97% of the South Korean population. The NHIS provides biannual health check-ups for all the insured citizens. These include a questionnaire concerning the past medical history and health-related behaviors (smoking and drinking), anthropometric measurements (e.g., BMI, blood pressure), and laboratory test findings (e.g., fasting glucose, lipid levels)^25^. The database contains extensive data from 50,000,000 Koreans encompassing patient demographics, medical treatments and procedures, and disease diagnoses according to the International Classification of Disease-10th Revision-Clinical Modification (ICD-10-CM) codes. Since 2015, the South Korean NHIS has released a nationally representative data set that includes nearly the entire South Korean population and is available to all researchers whose study protocols are approved by an official review committee. This study was approved by the NHIS inquiry commission, and the institutional review board of the Korea University Ansan Hospital (No. 2019AS0022).

**Figure 1:**
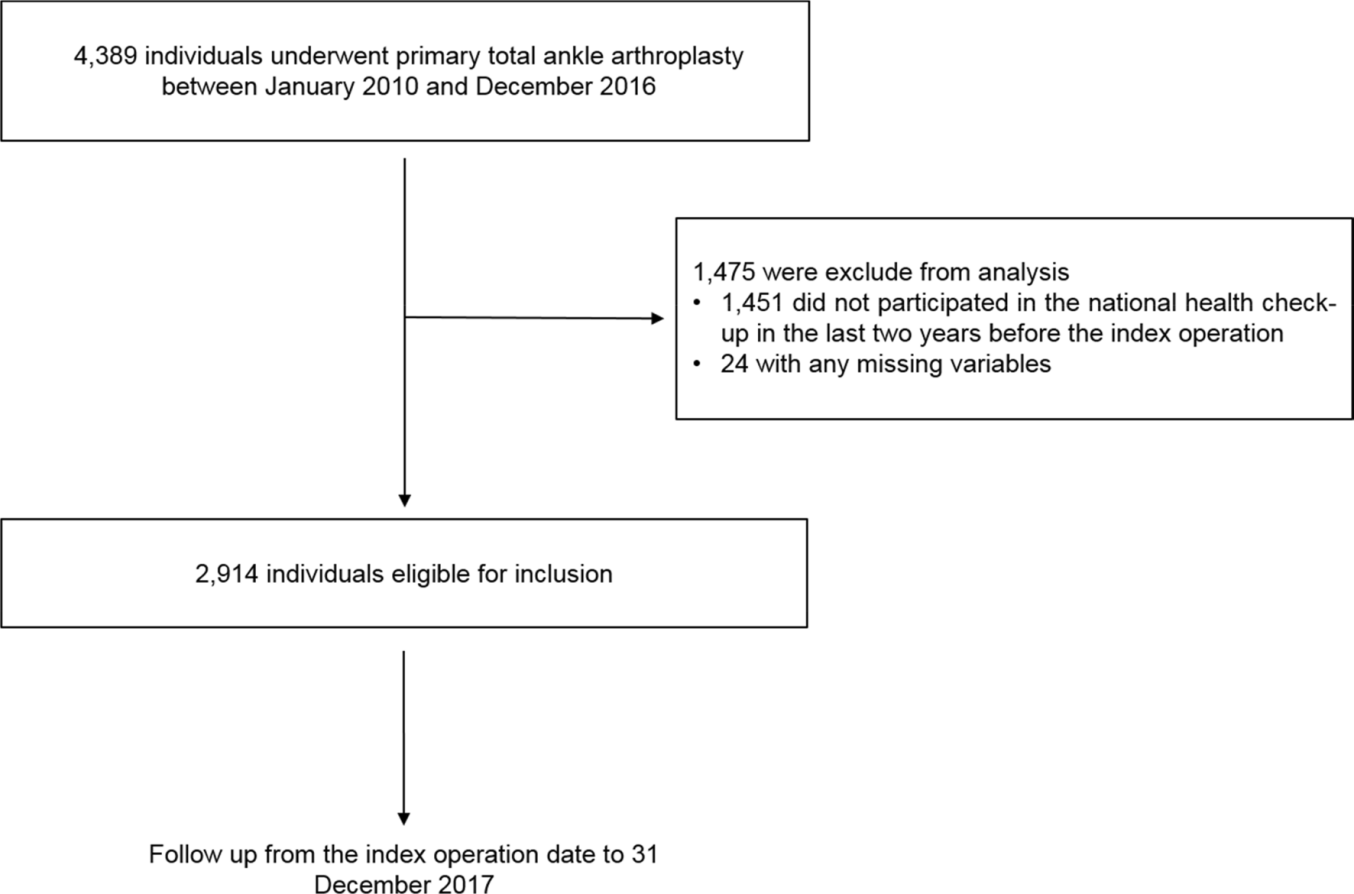
Flowchart of the study population.

We identified all primary TAAs performed in the country, that were defined by the claim codes N2075 and N2079, between January 1, 2010 and December 31, 2016. Only patients who underwent regular health check-ups in the last two years before the index TAA operation were included in the study. Subjects with missing data on the variables included in the statistical analysis were excluded. Altogether, 2,914 subjects were enrolled in this study (Fig. 1). The study endpoint was development of TAA failure at any point of time from the index operation date until 31 December 2017. The study population was followed from the baseline (the date of the index TAA operation) to the development of TAA failure or the end of 2017.

### Definition of TAA failure

The failure of TAA was defined as requiring revision TAA or arthrodesis procedures (i.e. AA and tibiotalocalcaneal arthrodesis). Revision TAA was identified by the claim codes N3715, N3729, N4715, and N4719. Arthrodesis procedures were identified by the claim codes N0733, N0735 and N0736, and N0737.

### Age and BMI categories

Participants were classified into seven age groups: age < 50 years, 50–54 years, 55–59 years, 60–64 years, 65–69 years, 70–74 years, age ≥ 75 years. Height and weight were measured during the health examinations. BMI (kg/m^2^) was calculated as weight in kilograms divided by the square of the height in meters. The World Health Organization recommendations for Asian populations were used to categorize individuals into five BMI groups: < 18.5 kg/m^2^ (underweight), 18.5–22.9 kg/m^2^ (normal), 23.0–24.9 kg/m^2^ (overweight), 25.0–29.9 kg/m^2^ (obese), and ≥ 30 kg/m^2^ (severely obese)^26^.

### Covariates

We assessed subject demographics and lifestyle factors through standardized self-reporting questionnaires. Smoking status was classified into non-, ex-, and current smoking categories. Alcohol consumption was divided into three categories: heavy drinker (more than 30 g/day of alcohol), mild drinker (less than 30 g/day of alcohol), and nondrinker (no alcohol at all)^27,28^. Regular physical activity was defined as performing strenuous exercise for at least 20 min three times per week or moderate exercise for at least 30 min five times per week. Income level was dichotomized at the lowest 20%. Blood pressure (BP) and serum levels of glucose, lipid profile, and creatinine were measured after overnight fasting. Baseline comorbidities, such as hypertension and dyslipidemia, were identified based on the combination of the medical history, health examination results, or ICD-10-CM and prescription codes. We defined hypertension as BP ≥ 140/90 mmHg, or at least one claim/year for an antihypertensive medication prescription under ICD-10-CM codes of I10-I13, I15. Diabetes mellitus (DM) was defined as fasting glucose ≥ 126 mg/dL, or at least one claim/year for an antidiabetic medication prescription under ICD-10-CM codes of E11-E14. Dyslipidemia was defined as a total cholesterol level ≥ 240 mg/dL or at least one claim per year for lipid-lowering medication (ICD-10-CM code E78). Hospital volume was categorized into five groups on the basis of the annual number of primary total ankle arthroplasties performed in each institute during the study period: < 10, 10–19, 20–29, 30–39, ≥ 40 primary procedures/year.

### Statistical analysis

The baseline characteristics were presented as the mean ± standard deviation or as a number with percentage. The Chi-square test was applied for categorical variables and Student’s t test for continuous variables to compare the characteristics between the ‘Non-failure’ and ‘Failure’ groups. The incidence rate of TTA failure was calculated from the number of incident cases divided by the follow-up duration in person-years. The cumulative incidence of TTA failure during follow-up according to age and BMI categories was assessed by Kaplan–Meier curves; the differences among the groups were evaluated using the log-rank test. Cox proportional hazard regression analysis was used to evaluate the association of age and BMI with TAA failure, and hazard ratios (HRs) and 95% confidence interval (CIs) were calculated. The results were adjusted for confounders including age, sex, BMI, hospital volume, income, DM, hypertension, dyslipidemia, and health-related behaviors (physical activity, smoking, and alcohol consumption). Statistical analyses were performed using SAS version 9.4 (SAS Institute, Cary, NC, USA). A 2-sided *P* value of 0.05 was considered statistically significant.

## Results

### Baseline characteristics

A comparison of the baseline characteristics between the non-failure and failure groups is presented in Table 1. A total of 2914 subjects who underwent primary TAA were included in this study. The failure of TAA occurred in 248 participants (8.5%) during the study period. The mean age (62.24 ± 8.26 years) of the failure group was significantly less than that (65.08 ± 8.29 years) of the non-failure group (*P* < 0.0001). The mean BMI (25.99 ± 3.65 kg/m^2^) of failure group was significantly greater than that (25.42 ± 3.18 kg/m^2^) of the non-failure group (*P* = 0.0079). There were no statistically significant differences between the two groups with regard to sex, income level, comorbidities such as DM, hypertension, and dyslipidemia, health-related behaviors (physical activity, smoking, and alcohol consumption), hospital volume, and cardiometabolic parameters such as systolic and diastolic BP, total cholesterol, and fasting plasma glucose.

**Table 1:** Baseline characteristics of study population.

	Non-failure group (*n* = 2666)	Failure group (*n* = 248)	*P* value
Male sex, *n* (%)	1418 (53.19)	125 (50.4)	0.4006
Age (years), mean (SD)	65.08 ± 8.29	62.24 ± 8.26	< 0.0001
**Age (years)**			0.0001
< 50	106 (3.98)	15 (6.05)	
50–54	221 (8.29)	28 (11.29)	
55–59	303 (11.37)	41 (16.53)	
60–64	560 (21.01)	68 (27.42)	
65–69	576 (21.61)	43 (17.34)	
70–74	624 (23.41)	41 (16.53)	
≥ 75	276 (10.35)	12 (4.84)	
BMI (kg/m^2^), mean (SD)	25.42 ± 3.18	25.99 ± 3.65	0.0079
**BMI (kg/m**^**2**^**)**			0.0543
< 18.5	25 (0.94)	2 (0.81)	
18.5–22.9	557 (20.89)	49 (19.76)	
23.0–24.9	632 (23.71)	56 (22.58)	
25.0–29.9	1237 (46.4)	107 (43.15)	
≥ 30	215 (8.06)	34 (13.71)	
**BP (mmHg), mean (SD)**			
Systolic	129.74 ± 15.21	129.6 ± 15.43	0.8919
Diastolic	79.21 ± 9.73	79.17 ± 10.69	0.9513
Fasting glucose (mg/dL), mean (SD)	104.7 ± 28.67	101.08 ± 21.93	0.0532
Total cholesterol (mg/dL),	197.98 ± 40.53	196.73 ± 38.48	0.6396
**Smoking status, n (%)**			0.1683
Non-smoker	1808 (67.82)	182 (73.39)	
Ex-smoker	474 (17.78)	39 (15.73)	
Current smoker	384 (14.4)	27 (10.89)	
**Alcohol consumption, n (%)**			0.8849
None	1752 (65.72)	162 (65.32)	
Mild	701 (26.29)	64 (25.81)	
Heavy	213 (7.99)	22 (8.87)	
Regular exerciser, *n* (%)	542 (20.33)	63 (25.4)	0.0596
Household income (lower 20%), *n* (%)	498 (18.68)	48 (19.35)	0.7944
**Comorbidities, n (%)**			
Diabetes mellitus	674 (25.28)	60 (24.19)	0.7058
Hypertension	1819 (68.23)	174 (70.16)	0.5314
Dyslipidemia	1285 (48.2)	117 (47.18)	0.758
**Hospital volume, procedures per year**			0.8442
< 10	1528 (57.31)	139 (56.05)	
10–19	398 (14.93)	42 (16.94)	
20–29	194 (7.28)	20 (8.06)	
30–39	172 (6.45)	13 (5.24)	
≥ 40	374 (14.03)	34 (13.71)	

### Incidence and risk of TAA failure according to age and BMI

**Figure 2:**
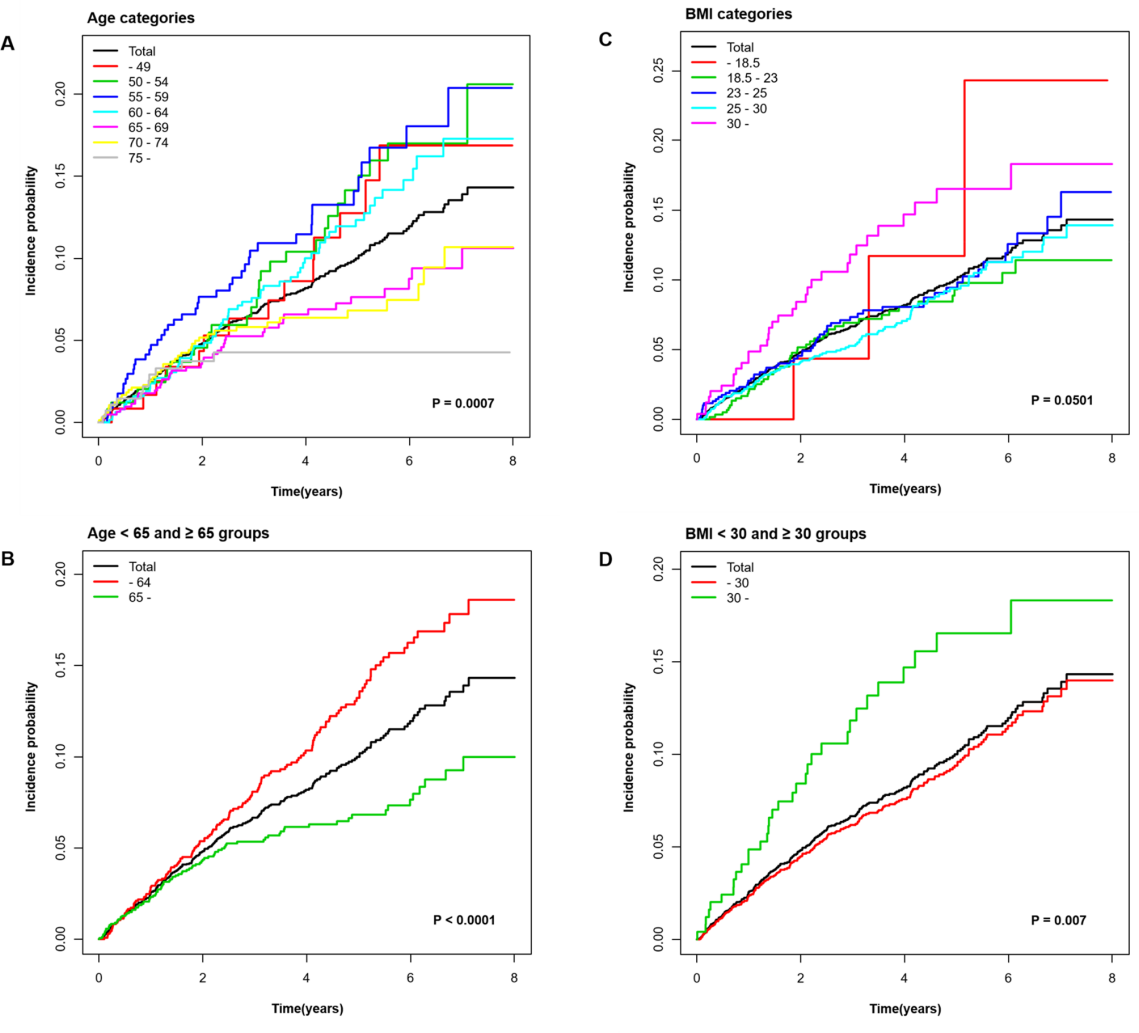
Cumulative incidence of TAA failure during follow‑up according to age categories (**A**), age < 65 and ≥ 65 groups (**B**), BMI categories (**C**), and BMI < 30 and ≥ 30 groups. The log‑rank test was applied to evaluate differences among the groups and calculate the *P* values.

During a mean follow-up of 3.82 ± 1.99 years (11,144 person-years), 248 TAA failure developed. The cumulative incidence of TAA failure is presented according to age and BMI categories using Kaplan–Meier curves (Fig. 2). The incidence rate of TAA failure significantly differed between age categories (*P* = 0.0007) and was significantly higher in the age < 65 group than that of age ≥ 65 group (*P* < 0.0001). The incidence rate of TAA failure did not differ significantly between BMI categories (*P* = 0.0501) but was significantly higher in the BMI ≥ 30 group than that of BMI < 30 group (*P* = 0.0037).

**Figure 3:**
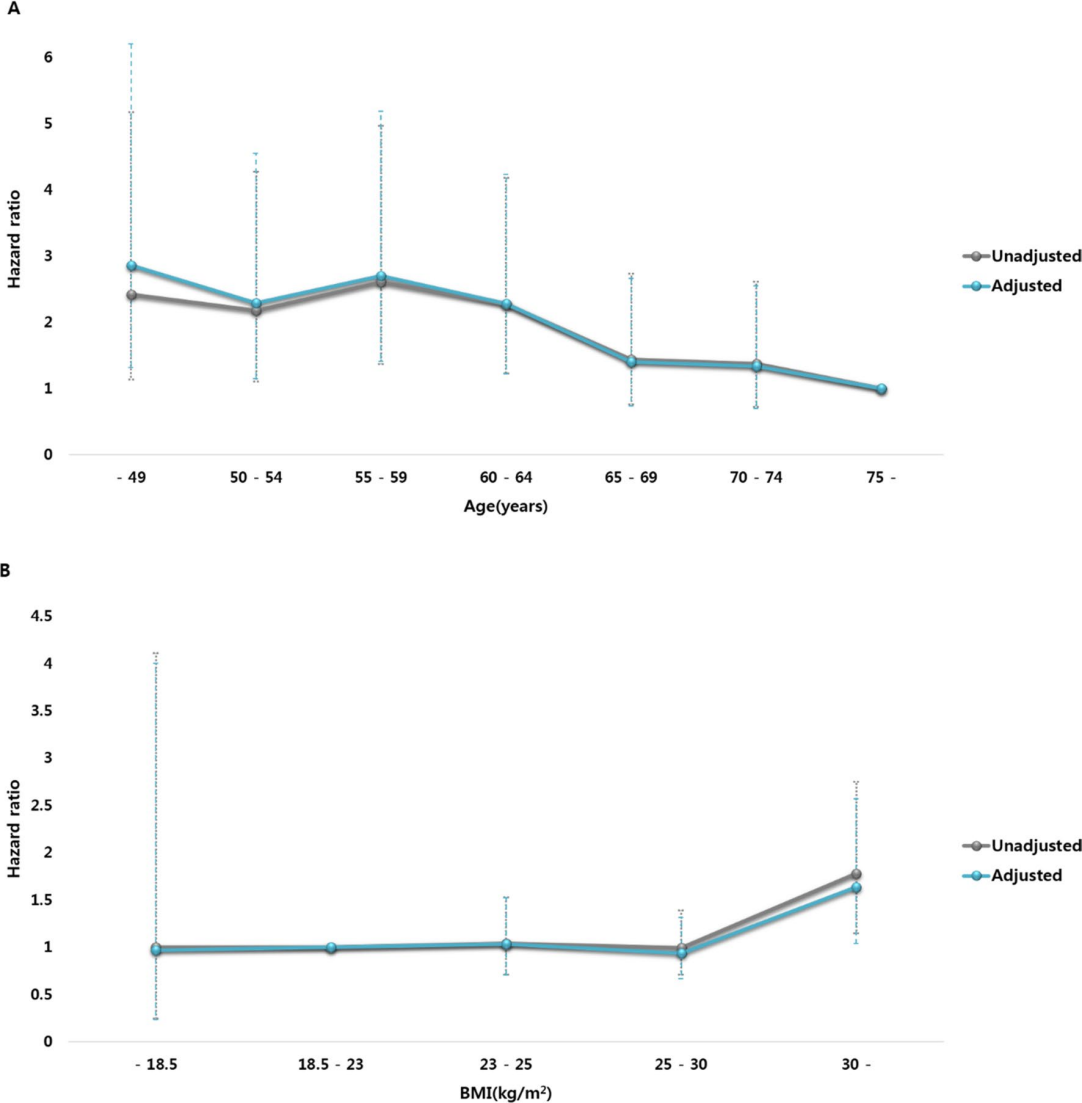
HR (95% CI) of TTA failure according to age (**A**) and BMI (**B**) categories.

The HRs (95% CIs) of TAA failure according to age and BMI categories are presented in Table 2. An increase of age by one year was significantly associated with 3.5% decreased HR for TAA failure. An increased risk of TAA failure was observed in the age < 65 group versus age ≥ 75 group [adjusted hazard ratio (aHR) 2.273, 95% CI 1.223–4.226 in the 60–64 age group; aHR 2.697, 95% CI 1.405–5.178 in the 55–59 age group; aHR 2.281, 95% CI 1.145–4.543 in the 50–54 age group; aHR 2.851, 95% CI 1.311–6.203 in the < 50 age group; Fig. 3A]. Conversely, age ≥ 65 group displayed no increase in the risk of TAA failure (aHR 1.394, 95% CI 0.732–2.656 in the 65–69 age group; aHR 1.334, 95% CI 0.700–2.543 in the 70–74 age group).

**Table 2:** Age, BMI, and the risk of TAA failure.

Variables	N	TAA failure	Person-years (PYs)	Incidence Rate (per 1,000 PYs)	Crude HR (95% CI)	Adjusted HR* (95% CI)
Age (per 1 year)					0.970 (0.957, 0.984)	0.965 (0.950, 0.980)
**Age (years)**						
< 50	121	15	511.61	29.32	2.420 (1.132, 5.172)	2.851 (1.311, 6.203)
50–54	249	28	1064.68	26.30	2.172 (1.104, 4.275)	2.281 (1.145, 4.543)
55–59	344	41	1279.21	32.05	2.605 (1.369, 4.958)	2.697 (1.405, 5.178)
60–64	628	68	2451.21	27.74	2.263 (1.225, 4.182)	2.273 (1.223, 4.226)
65–69	619	43	2454.72	17.52	1.437 (0.757, 2.726)	1.394 (0.732, 2.656)
70–74	665	41	2424.44	16.91	1.371 (0.720, 2.609)	1.334 (0.700, 2.543)
≥ 75	288	12	958.13	12.52	1 (ref.)	1 (ref.)
BMI (per 1 kg/m^2)^					1.051 (1.012, 1.091)	1.042 (1.002, 1.083)
BMI (kg/m^2^)						
< 18.5	27	2	94.75	21.11	1.000 (0.243, 4.113)	0.964 (0.232, 4.002)
18.5–22.9	606	49	2363.14	20.74	1 (ref.)	1 (ref.)
23.0–24.9	688	56	2579.06	21.71	1.040 (0.709, 1.527)	1.034 (0.703, 1.521)
25.0–29.9	1344	107	5191.26	20.61	0.991 (0.707, 1.389)	0.934 (0.663, 1.316)
≥ 30	249	34	915.79	37.13	1.775 (1.146, 2.749)	1.632 (1.036, 2.570)

*Adjusted for age, sex, BMI, hospital volume, income, diabetes mellitus, hypertension, dyslipidemia, and health-related behaviors (physical activity, smoking, and alcohol).

BMI, body mass index; TAA, total ankle arthroplasty; HR, hazard ratio; 95% CI 95% confidence interval.

BMI increase by every 1 kg/m^2^ was significantly associated with 4.2% increased HR for TAA failure and the risk was increased in the severely obese group versus the normal BMI group (aHR 1.632, 95% CI 1.036–2.570; Fig. 3B). There was no decrease in the risk in the underweight group (aHR 0.964, 95% CI 0.232–4.002).

## Discussion

Even though the popularity of TAA is growing for the treatment of end-stage ankle arthritis along with advances in implant design and surgical techniques, the complication and reoperation rates after primary TAA still remain high compared with those for AA^3,29^. Consequently, numerous studies have focused on identification of the risk factors for TAA failure. However, there are few large studies that have evaluated the risk factors for TAA failure. Furthermore, the reported risk factors have differed between studies^30^. We sought to conduct a nationwide population-based cohort study to identify the risk factors for implant failure after primary TAA.

Majority of the studies reported that the age at the time of initial TAA surgery was predictive of failure, with younger patients having a higher likelihood of requiring revision, although the definition of implant failure and the type of implant varied from study to study^5,18,19,31^. In a study of 684 patients (722 ankles) using the HINTEGRA prosthesis, age less than 70 years was identified as an independent risk factor for implant failure^5^. In another study with 72 patients (77 ankles) using the STAR prosthesis, the patients who underwent component revision were significantly younger at the time of total ankle replacement (50.4 ± 10.0 years) than those without revision surgery (57.1 ± 14.5 years)^18^. A multivariate regression analysis in 115 patients based on the use of the Agility prosthesis demonstrated that patient age at the time of surgery was predictive of implant failure^19^. Conversely, in a prospective study with a database of 538 ankle replacements, four implants (INBONE I, INBONE II, STAR, and Salto-Talaris) were used and age was not associated with higher failure rates in a multivariable logistic regression analysis^30^. A retrospective study^32^ of 811 HINTEGRA total ankles reported that clinical outcomes and the probability of revision surgery after TAA were comparable between young and old patients; the risk of revision surgery is not affected by age.

The impact of obesity on the outcomes after TAA is inconclusive. In a retrospective study^33^ of 97 ankle replacements based on the use of four implants (Agility LP, Agility, INBONE I, and Salto-Talaris), patients were separated into a reference group with a BMI less than 30 kg/m^2^ and an obese group with a BMI greater than or equal to 30 kg/m^2^. A multivariable logistic regression analysis demonstrated that obese patients had a significantly higher probability of implant failure by the final follow-up date [adjusted odds ratio (OR) 2.8, 95% CI 1.04–7.53]. Similarly, in a study using a large database of 905 patients who underwent TAA, BMI (OR 1.04, *P* = 0.046) was an independent risk factor for early adverse events following TAA^31^. Conversely, in a retrospective study involving 684 patients (722 ankles) using the HINTEGRA prosthesis, BMI was not associated with failure of the prosthesis in a univariate Cox regression analysis^5^. In a prospective study with a database of 538 ankle replacements, BMI was not associated with higher failure rates in a multivariable logistic regression^30^. A retrospective study of 115 patients with the use of the Agility prosthesis reported no correlation between BMI and prosthesis failure, with the average BMI in the survival group being similar to that in the failure group (28.3 vs 29.4; *P* = 0.27)^19^.

Despite the fact that DM negatively affects clinical outcomes and perioperative complications following TAA, a review of the literature reveals that diabetes has no correlation with implant failure following TAA^19,22,23,30^. A retrospective study involving 173 patients who underwent TAA with the HINTEGRA prosthesis reported that DM, especially when associated with poor glycemic control, negatively affects the short- to mid-term outcome after TAA but there was no significant difference between the non-diabetic and diabetic groups regarding the TAA revision^22^. A retrospective study of 115 patients with the use of the Agility prosthesis, also reported no significant difference between the non-diabetic and diabetic groups with regard to TAA failure^19^. Additionally, a retrospective cohort study involving 538 ankle replacements demonstrated that DM was not associated with implant failure in a univariable analysis^30^.

Few studies have investigated the effect of cigarette smoking on TAA failure. In a retrospective study of 646 TAA using the INBONE I, STAR, and Salto-Talaris prostheses, active cigarette smokers had a significantly higher risk of wound complications and worse outcomes than nonsmokers and former smokers^21^. However, active smoking did not significantly increase the rate of revision surgery. A retrospective study of 115 patients with use of the Agility prosthesis also found no correlation between TAA failure and smoking^19^.

Our most significant finding is that, firstly, age of less than 65 years was identified as an independent risk factor for TAA failure. Additionally, in the younger age groups there was a higher aHR for TAA failure. Secondly, BMI of ≥ 30 kg/m^2^ was independently associated with a statistically significant increase in the risk for TAA failure. Concerning the association between DM or cigarette smoking and TAA failure, we concluded that these factors were not associated with a higher risk of TAA failure, which is comparable to the results of previous studies^19,21,22,30^. The strengths of our study include the large sample size encompassing the entire South Korean population, longitudinal follow-up of up to 8 years, and extensive data from the regular health check-ups concerning demographics, socioeconomic factors, comorbidity, laboratory test results, and lifestyle variables.

However, the current study has several limitations. Firstly, the subjects who did not undergo a health check-up in the last two years before the index TAA operation were excluded, increasing the risk of a selection bias. Secondly, the data on health-related behaviors was reliant on self-reporting, which may be highly subjective and unreliable. Thirdly, the baseline comorbidities were defined by the health examination results or the health claim data, but not by the clinical records, and therefore could be subject to the risk of under- or over identification. However, we used the diagnosis code and medication records together, which has been shown to have higher accuracy^34^. Fourthly, all possible confounding factors, including multiple different diagnoses of ankle arthritis or the different types of implants were not considered. During the study, four implants, including HINTEGRA, Mobility, mobile-bearing Salto, and Zenith prostheses, were used in South Korea. However, we could not investigate the effect of implant type on TAA failure because information on implant type was not included in the database. Finally, the database provided by the South Korean NHIS does not include the medical records, thus we could not investigate the causes of TAA failure such as infection, aseptic loosening, osteolysis, preoperative deformity, or implant malalignment. Technical errors may also play a significant role in TAA failure. However, an analysis of technical errors was impossible in this study because we could not obtain clinical data from the database.

In conclusion, this population-based longitudinal study demonstrated that age < 65 years and BMI of ≥ 30 kg/m^2^ were associated with increased risk of TAA failure. Consequently, these factors should be considered when the surgeon and patient discuss possible surgical treatment options for end-stage ankle arthritis.
